# Serum 25-Hydroxyvitamin D Is Differentially Associated with Early and Late Age-Related Macular Degeneration in the United States Population

**DOI:** 10.3390/nu15051216

**Published:** 2023-02-28

**Authors:** Yihang Fu, Xiaoyun Chen, Sheng Luo, Shuangyan Jiang, Yuxiang Mao, Wei Xiao

**Affiliations:** State Key Laboratory of Ophthalmology, Zhongshan Ophthalmic Center, Sun Yat-sen University, Guangdong Provincial Key Laboratory of Ophthalmology and Visual Science, Guangzhou 510060, China

**Keywords:** nutrition, age-related macular degeneration, 25-hydroxyvitamin D, NHANES, RCS analyses

## Abstract

Background: Age-related macular degeneration (AMD) has been the leading cause of irreversible blindness in industrialized countries. Emerging data suggest that serum vitamin D levels may be associated with AMD but show mixed results. National-level population data on the relationship between vitamin D and AMD severities are still lacking. Methods: We used data from the National Health and Nutrition Examination Survey (NHANES) 2005 to 2008. Retinal photographs were taken and graded for AMD stage. The odds ratio (OR) of AMD and its subtype was calculated after adjusting for confounding factors. Restricted cubic spline (RCS) analyses were used to explore potential non-linear relations. Results: A total of 5041 participants with a mean age of 59.6 years were included. After adjusting for covariates, participants with higher level of serum 25-hydroxyvitamin D [25(OH)D] had significantly greater odds of early AMD (OR, 1.65; 95% CI, 1.08–2.51) and decreased risk of late AMD (OR, 0.29; 95% CI, 0.09–0.88). When stratified by age, a positive association between the level of serum 25(OH)D and early AMD was present in the <60 years group (OR, 2.79; 95% CI, 1.08–7.29), whereas a negative relation between the level of serum 25(OH)D and late AMD was detected in the ≥60 years group (OR, 0.24; 95% CI, 0.08–0.76). Conclusions: A higher level of serum 25(OH)D was related to increased risk of early AMD in those <60 years and decreased risk of late AMD in those ≥60 years.

## 1. Introduction

Age-related macular degeneration (AMD) is the leading cause of irreversible blindness among people beyond the age of 50 in industrialized countries [1,2]. Wong et al. reported a prevalence of 8.7% of AMD worldwide in 2014 and estimated that 288 million people would be affected in 2040 [3]. Clinically, AMD can be divided into early and late stage. The early stage is characterized by the presence of drusen and pigmentary changes in the macular area, and the late stage can be dry (also known as non-exudative, atrophic, or non-neovascular) or wet (known as exudative or neovascular) [4]. Featured with the choroidal neovascularization complex, wet AMD can result in leakage of blood and progresses rapidly in weeks or months. Dry AMD, however, which involves the loss of choriocapillaris and the retinal pigment epithelium, shows slower progress [4,5]. Though less common, late AMD can substantially decrease the quality of life and disrupt daily activities through affecting reading and computer use, driving, face recognition, etc., causing loss of independence in retirement years [6,7].

As a kind of secosteroid hormone, vitamin D is similar to other steroid hormones in structure but with an open B-ring [8]. It takes diversified forms due to different chemical structures of side chains. Vitamin D_3_ (cholecalciferol) and vitamin D_2_ (ergocalciferol) are two major precursor molecules of it. As the natural form of vitamin D, the former derives from fish oils, eggs, and animal fats, while vitamin D_2_ is found in irradiated plants. Except when obtained in the diet, vitamin D can also be synthesized in the skin, which is the most significant source. Specifically, 7-dehydrocholesterol produces pre-vitamin D_3_ under irradiation by ultraviolet B (UVB, λ = 290–315 nm) then forms vitamin D_3_ through temperature-sensitive rearrangement in the skin. After binding to vitamin D-binding protein (DBP) and being released into the bloodstream, vitamin D_3_ is converted to 25-hydroxyvitamin D_3_ [25(OH)D_3_] in the liver by two 25-hydroxylases, cytochrome P-450 enzyme (CYP) CYP2R1 and CYP27A1 [9]. Then, 25(OH)D_3_ is transported to the kidney and metabolized to 1,25-dihydroxyvitamin D_3_ [1,25(OH)_2_D_3_] by 1α hydroxylase, CYP27B1 [9]. Though 1,25(OH)_2_D_3_ is the functionally active form of vitamin D, 25(OH)D_3_ is the major circulating form owing to its longer half-life (about 3 weeks) [10]. Ultimately, both 25(OH)D_3_ and 1,25(OH)_2_D_3_ can be hydroxylated at C-24 by the deactivating enzyme CYP24A1 in the kidney and thus downregulated [11]. Vitamin D not only plays a critical role in maintaining the normal function of musculoskeletal, immune, and nervous systems, but has a great impact on mineral metabolism homeostasis, gene expression, cell proliferation and differentiation, and angiogenesis [12,13,14].

The pathogenesis of AMD is regarded as multifactorial, comprising a complex interaction between aging, genetic susceptibility, behavioral, and environmental risk factors. As a nutrient with diverse biological functions, numerous studies have documented the vital roles of vitamin D in maintaining ocular as well as visual health [14]. Experimental studies have revealed that vitamin D can block several key pathological processes underlying AMD, such as oxidative stress, inflammatory responses in retinal pigment epithelium (RPE) cells, and retinal neovascularization [8,15,16,17]. Serum vitamin D has been reported to be related to AMD in several clinical and epidemiology studies, but contradictory results have been obtained. Parekh et al. conducted the first study to examine the protective effect of vitamin D on AMD using the data from the third National Health and Nutrition Examination Survey III (NHANES III) and found that serum vitamin D level was negatively related to early but not late AMD [18]. This conclusion was further supported by the Atherosclerosis Risk in Communities cohort study [19]. Nevertheless, findings from the Korean population were fairly opposite: a high level of blood vitamin D was inversely associated with late AMD but was rather directly associated with early AMD [20,21]. Such discrepancies, largely caused by heterogeneity in study procedures, made pooling existing data difficult and drew no definitive association between serum vitamin D and AMD risk [22,23]. Moreover, all previous studies estimated risk of AMD by vitamin D level through establishing logistic regression models, which assume a linear relationship between the independent variable and the logit probability of positive outcome. However, recent studies increasingly demonstrated that serum vitamin D was associated with many outcomes in a non-linear relationship, for instance, an inverted U-shaped relationship with eczema and an L-shaped association with mortality [24,25].

NHANES is a large, continuous, cross-sectional series of surveys conducted by selecting a nationally representative sample of the resident civilian noninstitutionalized US population. In this study, we aim to examine the relationship between serum 25-hydroxyvitamin D [25(OH)D] levels and the odds of different subtypes of AMD with a focus on exploring their non-linear relationship, using the data of the NHANES 2005–2006 and 2007–2008 cycles.

## 2. Materials and Methods

### 2.1. Sample and Population

NHANES is a population-based cross-sectional program conducted by the National Center for Health Statistics (NCHS). The study protocol was reviewed and approved by the NCHS Research Ethics Review Board. Detailed methodology and data files are publicly accessible online [26]. Each cycle of the survey contains an interview component with demographic, socioeconomic, dietary, and health-related questions and an examination component comprising medical, dental, and physiological measurements and laboratory tests. For this study, survey data from the 2005–2006 and 2007–2008 cycles were obtained. Only participants with data on serum 25(OH)D, AMD, and sufficient covariates were considered eligible.

### 2.2. Age-Related Macular Degeneration Grading

In the 2005–2006 and 2007–2008 NHANES cycles, the Retinal Imaging subsection of the Ophthalmology Component tested for the presence of major retinopathies. For each eye, two 45-degree non-mydriatic digital retinal images were obtained by an ophthalmic digital imaging system. Digital retinal images were evaluated by graders at the University of Wisconsin. AMD was graded and staged according to the University of Wisconsin Age-Related Maculopathy Grading System [27]. Early AMD was defined as (1) soft drusen present with a grid area of greater than a 500 μm circle and a pigmentary abnormality present (increased pigment or depigmentation in the grid) or (2) soft drusen present in the center circle and a pigmentary abnormality present (increased pigment or depigmentation in the grid). Late AMD was defined as the presence of any late lesions, including geographic atrophy, pigment epithelial or retinal detachments, subretinal hemorrhage, subretinal fibrous scar, subretinal new vessels, or laser treatment and/or photodynamic therapy for AMD. All images were graded by at least two graders (a preliminary grader and a detail grader). If they disagreed, an adjudicator would evaluate the images to make a final decision. In this analysis, we selected the eye with a more severe AMD level when retinal photographs were gradable for both eyes.

### 2.3. Measurement of Vitamin D

For the 2005–2006 cycle, serum 25(OH)D concentrations were measured using the DiaSorin RIA kit (Stillwater, MN). Raw data were converted to equivalent 25(OH)D measurements by using a standardized liquid chromatography-tandem mass spectrometry (LC-MS/MS) regression formula.

LC-MS/MS _equivalent_ = 8.36753 + 0.97012*RIA _original_

After standardization, the 2005–2006 serum 25(OH)D data were comparable to those from the 2007–2008 cycle, which were directly obtained using the ultra-high-performance LC-MS/MS method.

According to the Institute of Medicine standard (referred to as ‘IOM standard’), serum 25(OH)D level was divided into three categories: deficient (<30 nmol/L), insufficient (30–50 nmol/L), and sufficient (>50 nmol/L) [28]. In addition, we utilized quintiles of 25(OH)D level to examine the relationship between 25(OH)D and AMD.

### 2.4. Covariates

The in-person interview data were used to extract the demographic data, lifestyle data, and medical history data. The demographic variables included age, gender, race/ethnicity, education (9th grade; 9–11th grade; high school graduate/GED or equivalent; college or AA degree; college graduate or above), and poverty–income ratio (PIR) (<130%; 130–349%; ≥350%).

Lifestyle variables included obesity, smoking status, and alcohol consumption. Obesity was categorized based on body mass index (BMI) as underweight (<18.5 kg/m^2^), normal (18.5–25.0 kg/m^2^), overweight (25.0–30.0 kg/m^2^), and obese (≥30.0 kg/m^2^). Smoking status was categorized as non-smokers (<100 cigarettes in their lifetime), past smokers (≥100 cigarettes in their lifetime, but had quit smoking at the time of interview), and current smokers (≥100 cigarettes in their lifetime, and currently smoke cigarettes). Alcohol consumption was categorized as never, former, and current drinker. Never drinkers were those who answered “no” to drinking any alcohol in their entire lifetime and in the past 12 months. Interviewees who answered “yes” to drinking in their entire lifetime but had not consumed alcohol in the past 12 months were coded as “former drinkers”. Current drinkers were defined as those had drunk at least 12 alcoholic drinks in the past year or in their entire lifetime and had consumed alcohol on at least one day in the past year.

Medical data consisted of information obtained from both a physical examination and a questionnaire. Self-reported general health condition was dichotomized as excellent, very good or good, fair or poor. History of cardiovascular disease (CVD) was defined as the presence of any of the following conditions: congestive heart failure, coronary heart disease, angina, heart attack, and stroke. Diabetes was defined as a self-reported diagnosis of diabetes by doctors, use of insulin or oral diabetic tablets, fasting glucose ≥ 7.0 mmol/L, or glycated hemoglobinA1c (HbA1c) ≥ 6.5%. Hypertension was defined as systolic blood pressure ≥ 130 mmHg or diastolic blood pressure ≥ 80 mmHg based on the mean value of 3 measurements or a self-declared history of hypertension or taking blood pressure medications [29,30]. Hypercholesterolemia was defined as a total cholesterol of 240 mg/dL (6.2 mmol/L) or higher or use of lipid-lowering medications. The history of ocular conditions was extracted from personal interview data on several vision topics. Cataract operation was identified by answers to the question: “Have you/Has standard patient (SP) ever had a cataract operation?” Glaucoma was determined by an affirmative answer to the question: “Has an ophthalmologist ever told you or SP that you have or he/she has glaucoma, sometimes called intraocular hypertension?”.

### 2.5. Statistics Analysis

We used R statistical package (R Core Team, Vienna, Austria, version 4.2.1) and STATA (Stata Corp, College Station, TX, USA, version 15.0) for all data analyses. Descriptive statistics were reported as means (standard deviations, SDs) for continuous variables and numbers (percentages) for categorical variables. To compare general characteristics of participants, we used the unpaired *t*-test for continuous variables and the Pearson *χ*^2^ test for categorizing data. To estimate odds ratios (ORs) of AMD according to serum 25(OH)D level, we established logistic regression models adjusting for demographic variables (age, sex, race, education, poverty–income ratio), lifestyle variables (BMI, smoking, drinking), and medical history variables (CVD, hypertension, diabetes, cataract surgery, glaucoma). The ORs and 95% confidence intervals (CIs) were calculated. Regression analysis was also stratified by age (≥60 years versus <60 years subgroups). To investigate non-linear relations, we used restricted cubic spline (RCS) logistic regression analyses with three knots to model the association between AMD subtypes and serum 25(OH)D level. Variables adjusted in multiple RCS models were the same as those in the most saturated logistic models. The R package rcssci was used to visualize splines [31]. ORs of 1.0 were set as the reference value. All *p*-values reported were 2-sided, and those less than 0.05 were considered statistically significant. We also consider *p* = 0.005, the threshold based on Bonferonni correction, when multivariate testing was simultaneously applied.

## 3. Results

### 3.1. General Characteristics of the Sample Population

**Figure 1:**
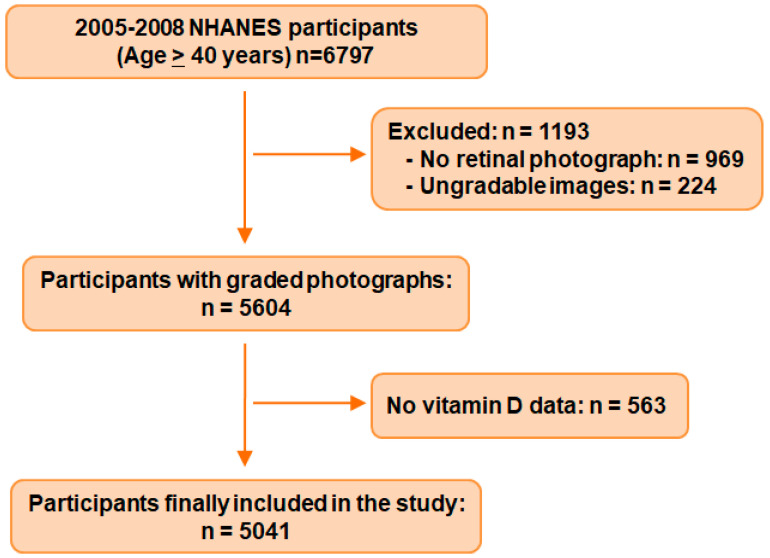
Flowchart of participant inclusion from NHANES 2005–2008.

Through combining the 2005–2006 and 2007–2008 NHANES data, we identified the initial sample of 6797 participants aged 40 years or older. We excluded 969 participants without retinal photographs for at least one eye and 224 with ungradable images. After ruling out 563 with missing data on serum 25(OH)D, a total of 5041 eligible participants were ultimately included in this analysis (Figure 1).

The general characteristics for subjects with versus without serum 25(OH)D data are shown in Table S1. Those with serum 25(OH)D data were older, more likely to be Mexican American or non-Hispanic white, former/current drinker, with better socioeconomic status, with better health status, and with hypercholesterolemia (all *p* < 0.05). There was no significant difference regarding AMD severity, gender, education, BMI, smoking status, and history of ocular condition (all *p* > 0.05, Table S1).

The general characteristics of the study population stratified by status of AMD are presented in Table 1. There were 405 participants (8.0%) with any AMD, including 354 with the early stage (7.0%) and 51 with the late stage (1.0%). The average concentration of serum 25(OH)D in the whole population was 60.1 ± 22.4 nmol/L. Mean serum 25(OH)D concentration was significantly higher in AMD individuals as compared to non-AMD ones (64.1 ± 22.3 nmol/L versus 59.8 ± 22.4 nmol/L; *p* < 0.001). In addition, AMD participants tend to be sufficient in serum 25(OH)D by IOM standard (*p* = 0.015). As expected, AMD participants were older, of non-Hispanic white race, with poorer socioeconomic status, and of past/current smoker status (all *p* < 0.05). Moreover, the AMD population had a higher prevalence of cardiovascular disease, hypertension, glaucoma, and history of cataract operation than their non-AMD counterparts (all *p* < 0.05). The differences in study cycle (2005–2006 vs. 2007–2008), gender, education, BMI, or general health condition were not significant between groups (all *p* > 0.05). When the Bonferroni correction was applied (*p*-value threshold = 0.005), variables including age, race, serum 25(OH)D level, PIR, smoking status, CVD, hypertension, and history of ocular conditions remain significantly different between the AMD and non-AMD groups.

### 3.2. Association of AMD and Serum 25(OH)D Level in the Whole Population

Table 2 summarizes AMD events according to serum 25(OH)D status. As shown, the rate of early AMD increased with the increasing level of the serum 25(OH)D. Specifically, the rate of early AMD in the deficiency group (<30 nmol/L) was 4.20%, whereas that of the sufficiency group (>50 nmol/L) increased to 7.92%. On the contrary, late AMD had an inverse relationship with the level of serum 25(OH)D. The rate of late AMD in the sufficiency group was lower than that of the deficiency group (1.06% versus 1.62%), and this trend was more apparent among patients over 60 years (2.06% versus 3.82%).

The results of multivariable logistic regression are shown in Table 3. Participants in the sufficiency 25(OH)D group (≥50 nmol/L) showed significantly higher odds of being diagnosed as having any and early AMD in crude (unadjusted) models (for any AMD: OR = 1.61; 95% CI, 1.03–2.51; for early AMD: OR = 1.96; 95% CI, 1.17–3.29). Such a relationship, however, disappeared after adjusting for demographic, lifestyle, and medical comorbidity variables (model 1 and model 2, all *p* > 0.05). The odds ratios showed no significant differences between the insufficiency group (30–50 nmol/L) in reference to the deficiency group in all models for any and early AMD. Nonetheless, both the insufficiency and sufficiency groups yielded significantly lower risks of having late AMD in the saturated model (model 2, for insufficiency: OR = 0.27; 95% CI, 0.08–0.96; for sufficiency: OR = 0.29; 95% CI, 0.09–0.88).

When we utilized quintile analysis (Table S2), it showed consistent results with those of IOM standard. Significantly, the lowest quintile group had the lowest prevalence in both any or early AMD among the overall population, and this situation also appeared in both the under and over 60 years groups. Moreover, compared with subjects with the lowest quintile group, those within the fourth quintile (64.2–78.0 nmol/L) displayed higher risk of any AMD in the crude model and of early AMD in all models. Likewise, those in the highest quintile demonstrated significantly higher ORs in crude models of any AMD as well as early AMD and in model 1 of early AMD (OR = 1.54, 95% CI, 1.00–2.35).

### 3.3. Association of AMD and Serum 25(OH)D Stratified by Age

Given the mean age of 59.6 years for the whole sample population, we divided all participants into the younger (<60 years) and older group (≥60 years). Table 4 displayed the results of multivariable logistic regression according to age subgroups.

In the <60 years population, no significant association was detected between any or early AMD and serum 25(OH)D under the IOM standard. Under quintile subcategories (Table S2), compared with the lowest quintile, quintiles 2 and 4 had higher odds of any AMD (model 2 of quintile 4: OR = 2.95, 95% CI: 1.14–7.68). Parallel trends were also found in quintiles 2 and 4 for early AMD (model 2 of quintile 4: OR = 2.79, 95% CI, 1.08–7.29). The association between late AMD and serum 25(OH)D in the younger subgroup was not analyzed due to the lack of sufficient cases with late AMD (Table 2 and Table 4).

In the ≥60 years population, there was no significant association between any or early AMD and serum 25(OH)D level under IOM standard, and the quintile analysis showed similar results (Table S2). As for late AMD, however, we discovered that both the insufficiency and sufficiency groups showed significantly lower ORs in model 2 for late AMD (OR = 0.26, 95% CI, 0.07–0.94, and OR = 0.24, 95% CI, 0.08–0.76, respectively) as compared with the deficiency group.

Moreover, the crude model of quintile analysis showed increasing risk of early AMD with increasing quintiles (Table S2). In addition, the highest two quintiles displayed higher risks of any AMD in the crude model (OR = 1.62, 95% CI: 1.07–2.44, and OR = 1.61, 95% CI: 1.07–2.43, respectively) but became insignificant after adjusting covariates (Table S2).

### 3.4. Restricted Cubic Spline Analysis

**Figure 2:**
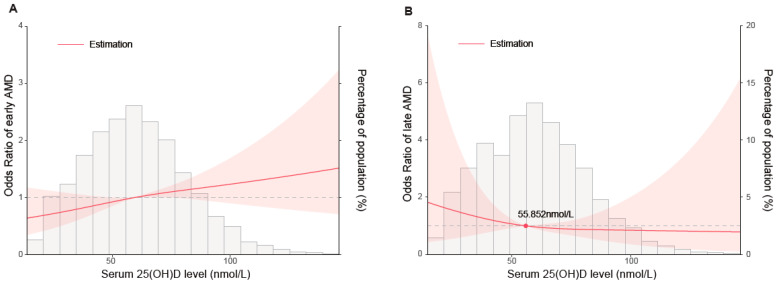
Restricted cubic spline analysis of serum 25(OH)D and odds ratio of early (**A**) and late AMD (**B**).

As the spline analysis presented, the risk of early AMD was positively associated with the level of serum 25(OH)D in an approximately linear pattern (Figure 2A). Despite the wide 95% confidence interval, lower levels of serum 25(OH)D were connected with a decreased risk of early AMD whereafter the risk continuously increased. On the other hand, the non-linear and L-shaped association between serum 25(OH)D level and late AMD was found (Figure 2B). The risk gradually decreased with the inflection point of 55.852 nmol/L. However, the 95% confidence interval included the odds ratio of 1.0 at any level of serum 25(OH)D for advanced AMD.

## 4. Discussion

In this study, we discovered that serum 25(OH)D had differential association with early and late AMD in the US population. Specifically, serum 25(OH)D level was positively associated with the risk of early AMD in a linear pattern, whereas it was negatively associated with late AMD, showing an L-shaped relationship. Intriguingly, a high serum 25(OH)D level was only related to increased odds of early AMD in adults aged <60 years, but it was related to a decreased risk of late AMD in those aged ≥60 years. These results indicated that maintaining serum 25(OH)D within certain levels may have the benefit of reducing the risk of early or late AMD for at-risk individuals.

Our unique findings add to the body of literature exploring the association between 25(OH)D level and AMD risks. Previous studies investigated this association but showed mixed results. In regard to early AMD, as our RCS analysis presented, there was an approximately positive linear pattern between serum 25(OH)D and early AMD, which means higher levels of serum 25(OH)D probably confer no benefits on early AMD. A previous NHANESIII (1988 through 1994) study found that higher serum vitamin D level was inversely associated with early AMD (OR = 0.64, 95% CI: 0.5–0.8) [18]. However, AMD was ascertained in merely one eye, probably leading to an underestimation of cases [27]. Thereafter, several cross-sectional studies explored this topic among different populations. One involving 968 postmenopausal women of the Carotenoids in Age-related Eye Disease Study (CAREDS) showed that higher serum 25(OH)D concentrations may protect against early AMD in women aged <75 years (OR = 0.52, 95% CI: 0.29–0.91) [32]. However, CAREDS was limited by the unfavorable representativeness of the sample population (i.e., US white and postmenopausal women rather than a national sample). In this study, we found an overall positive correlation between early AMD and serum 25(OH)D by quintiles. When stratified by age, such a correlation was only present in people aged 60 years or younger. Consistent with logistic analysis, the spline analysis showed that the risk of early AMD was positively associated with the level of serum 25(OH)D in an approximately linear pattern. The mechanism underlying such an association is to be examined. A possible explanation is that individuals with higher serum 25(OH)D may have undergone prolonged sunlight exposure, a factor posing risk to early AMD [33,34]. Clinically, our findings implied that 25(OH)D supplementation might not be beneficial but even harmful in reducing early AMD risk. Though we proposed several possible explanations why serum 25(OH)D was associated with a higher risk of early AMD, such findings are still logically opposite to the biological functions of vitamin D in health and eye conditions. These findings should be further confirmed in future studies.

Conversely, there was a significant inverse association between serum 25(OH)D level and late AMD in the whole population and the subpopulation aged 60 years or older. The spline analysis further revealed an L-shaped association between serum 25(OH)D and the risk of late AMD with the inflection point of 55.852 nmol/L. Such findings have been supported by most existing evidence. A meta-analysis aggregating nine cross-sectional studies showed a protective effect of high vitamin D concentrations against late AMD (pooled OR = 0.47, 95% CI: 0.28–0.79). This study also proposed a non-linear association, since the vitamin D threshold of 50 nmol/L was associated with late AMD, but there was no such association at 25 nmol/L [35]. A consistent result was also reported in an observational twin study: monozygotic twins with less severe AMD had higher vitamin D intake as compared to their co-twins [36]. These results were further confirmed by a prospective study which investigated the impact of dietary vitamin D intake on advanced AMD. In that study, higher dietary vitamin D intake could induce a 40% decrease in the risk of progression to advanced AMD (hazard ratio [HR]: 0.60; 95% CI: 0.43–0.83) [37]. The protective effect of 25(OH)D on late AMD could in part be explained from the perspective of biology: 25(OH)D can exert antiangiogenic and antioxidant properties [38,39], inhibit the transforming growth factor β pathway, and reduce the production of inflammatory markers associated with late AMD pathogenesis [40,41,42].

There are some conditions leading to vitamin D deficiency that could concurrently influence the severity of AMD. Firstly, based on the source of vitamin D, inadequate sun exposure and limited intake are two common reasons for decreased serum vitamin D level, as mentioned above [43]. Secondly, impaired intestinal absorption, such as inflammatory bowel disease (IBD), may also cause vitamin D deficiency, despite the fact that there is still no clear conclusion that it is a cause or a consequence of IBD [43,44]. However, IBD diseases including Crohn’s disease (CD) and ulcerative colitis (UC) are proven to be related to intestinal microbiota dysbiosis, which may play a crucial part in AMD development due to its ascertained impact on low-grade inflammation [45,46,47,48]. Moreover, as the liver is the center of vitamin D synthesis, several diseases, including liver cirrhosis, viral hepatitis, and fatty liver diseases, can result in vitamin D deficiency [49,50]. A previous cross-sectional study discovered that individuals with hepatitis B infection had significantly higher risk of AMD (OR = 2.566, 95% CI: 1.519–4.335) [51]. Bum-Joo et al. [52] proposed that a history of liver cancer was associated with a higher prevalence of both any and late AMD (OR = 4.32, 95% CI: 1.74–10.71; OR = 12.51, 95% CI: 1.39–112.87, respectively). Nevertheless, the mechanisms behind the association between AMD and liver diseases need to be further explored. Ultimately, kidney disease would also cause the decreased level of serum 1,25(OH)_2_D_3_ by impairing the activity of renal 1α hydroxylase [12]. Interestingly, studies showed that CKD and AMD not only share risk factors such as obesity, hypertension, and smoking, but also have a common pathophysiology mechanism as well as genetic predisposition [53,54,55,56]. Several previous studies have already found the association of the two conditions. A prospective study involving 1183 individuals reported that those with moderate CKD were three times more likely to have early AMD (OR = 3.2, 95% CI: 1.8–5.7) [57]. Another meta-analysis showed a positive relationship between CKD and AMD (OR = 1.35, 95% CI: 1.03–1.73) [58]. In addition, Amisha et al. [59] demonstrated that reduced kidney function was significantly associated with AMD by using NHANESIII data.

### Strengths and Limitations

The main strength of our analysis is that we used the restricted cubic spline (RCS) to examine the association between serum level of 25(OH)D and different stages of AMD. It is capable of summarizing a non-linear relationship between an outcome and the explanatory variable. Furthermore, the data we used were from the NHANES, which can desirably represent the general US adult population. In addition, we adjusted multiple potential confounders when constructing both binary and RCS logistic regression models. However, there were several limitations of our study as well. First, owing to the cross-sectional design, we were not able to establish a causal relationship between 25(OH)D and AMD. Second, as we had only one participant with late AMD aged less than 60 years old, we did not conduct subgroup analysis for the relation between late AMD and serum 25(OH)D. Moreover, other potential risk factors, such as time outdoors or sunlight exposure, may be relevant to the production of 25(OH)D and thus exert influence on developing AMD. Unfortunately, data on outdoor activity and sun exposure were not concurrently collected by NHANES from 2005 to 2008, so we are unable to directly answer whether outdoor time might have mediated the association between serum 25(OH)D level and the occurrence of AMD, especially for early stages.

## 5. Conclusions

In conclusion, we found a distinct association between serum 25(OH)D level and different types of AMD in US adults. A higher level of serum 25(OH)D was related to increased risk of early AMD in those aged 60 years or younger, but it was related to decreased risk of late AMD in individuals older than 60. More importantly, the association between serum 25(OH)D and late AMD was in a non-linear L-shaped pattern. Further prospective studies and randomized trials with long-term follow-up are needed to better evaluate the effectiveness of 25(OH)D on late AMD.

## Figures and Tables

**Table 1:** General characteristics of participants stratified by AMD status in NHANES, 2005–2008.

Variable	Participants without AMD	Participants with Any AMD	All Participants	*p*-Value
Sample size, n (%)	4636 (92.0)	405 (8.0)	5041	N.A.
Study cycle, n (%)				0.960
2005–2006	2158 (46.6)	188 (46.4)	2346 (46.5)	
2007–2008	2478 (53.4)	217 (53.6)	2695 (53.5)	
Age, years, mean (SD)	58.6 (12.1)	70.4 (11.4)	59.6 (12.5)	<0.001
Sex (female), n (%)	2310 (49.8)	194 (47.9)	2504 (49.7)	0.457
Race/ethnicity, n (%)				<0.001
Mexican American	738 (15.9)	48 (11.9)	784 (15.6)	
Other Hispanic	324 (7.0)	21 (5.2)	345 (6.8)	
Non-Hispanic White	2497 (53.9)	294 (72.6)	2791 (55.4)	
Non-Hispanic Black	933 (20.1)	33 (8.2)	966 (19.2)	
Other	144 (3.1)	9 (2.2)	153 (3.0)	
Serum 25(OH)D, nmol/L, mean (SD)	59.8 (22.4)	64.1 (22.3)	60.1 (22.4)	<0.001
25(OH)D status, n (%)				0.015
<30 (deficiency)	365 (7.9)	22 (5.4)	387 (7.6)	
30–50 (insufficiency)	1295 (27.9)	95 (23.5)	1390 (27.6)	
≥50 (sufficiency)	2976 (64.2)	288 (71.1)	3264 (64.8)	
Education, n (%)				0.297
Less than 9th grade	657 (14.2)	63 (15.6)	720 (14.3)	
9–11th grade	689 (14.9)	55 (13.6)	744 (14.8)	
High school grad/GED or equivalent	1134 (24.5)	113 (27.9)	1247 (24.7)	
Some college or AA degree	1180 (25.5)	106 (26.2)	1286 (25.5)	
College graduate or above	975 (21.0)	68 (16.8)	1043 (20.7)	
Unknown	1 (0.02)	0	1 (0.02)	
Poverty–income ratio, n (%)				<0.001
<130%	1073 (23.1)	95 (23.5)	1168 (23.2)	
130–349%	1625 (35.1)	183 (45.2)	1808 (35.9)	
≥350%	1655 (35.7)	90 (22.2)	1745 (34.6)	
Unknown	283 (6.1)	37 (9.1)	320 (6.4)	
Obesity, n (%)				0.059
Underweight	63 (1.4)	4 (1.0)	67 (1.3)	
Normal	1102 (23.8)	112 (27.7)	1214 (24.1)	
Overweight	1651 (35.6)	159 (39.3)	1810 (35.9)	
Obese	1784 (38.5)	127 (31.4)	1911 (37.9)	
Unknown	36 (0.8)	3 (0.7)	39 (0.8)	
Smoking status, n (%)				0.002
Non-smoker	2211 (47.7)	177 (43.7)	2388 (47.4)	
Past smoker	1480 (31.9)	165 (40.7)	1645 (32.6)	
Current smoker	943 (20.3)	63 (15.6)	1006 (20.0)	
Unknown	2 (0.04)	0	2 (0.04)	
Alcohol consumption, n (%)				0.027
Never drinker	612 (13.2)	75 (18.5)	687 (13.6)	
Former drinker	498 (10.7)	38 (9.4)	536 (10.6)	
Current drinker	3431 (74.0)	284 (70.1)	3715 (73.7)	
Unknown	95 (2.1)	8 (2.0)	103 (2.0)	
General health condition, n (%)				0.795
Excellent, very good, or good	3410 (73.6)	292 (72.1)	3702 (73.4)	
Fair, or poor	1146 (24.7)	105 (25.9)	1251 (24.8)	
Unknown	80 (1.7)	8 (2.0)	88 (1.8)	
History of systemic diseases, n (%)				
Cardiovascular disease	649 (14.0)	121 (29.9)	770 (15.3)	<0.001
Diabetes	952 (20.5)	81 (20.0)	1033 (20.5)	0.798
Hypertension	3055 (65.9)	309 (76.3)	3364 (66.7)	<0.001
Hypercholesterolemia	1910 (41.2)	174 (43.0)	2084 (41.3)	0.725
History of ocular condition, n (%)				
Cataract operation	502 (10.8)	131 (32.4)	633 (12.6)	<0.001
Glaucoma	256 (5.5)	42 (10.4)	298 (5.9)	<0.001

GED, general educational development. N.A., not applicable.

**Table 2:** Summary of AMD events according to serum 25(OH)D status.

Serum 25(OH)D (nmol/L)	Any AMD		Early AMD		Late AMD	
	No. At-Risk	No. with Event (%)	No. At-Risk	No. with Event (%)	No. At-Risk	No. with Event (%)
**Overall**						
<30 (deficiency)	387	22 (5.68)	381	16 (4.20)	371	6 (1.62)
30–50 (insufficiency)	1390	95 (6.83)	1377	82 (5.95)	1308	13 (0.99)
≥50 (sufficiency)	3264	288 (8.82)	3232	256 (7.92)	3008	32 (1.06)
**<60 years**						
<30 (deficiency)	217	3 (1.38)	217	3 (1.38)	214	0 (0)
30–50 (insufficiency)	753	23 (3.05)	753	23 (3.05)	730	0 (0)
≥50 (sufficiency)	1547	45 (2.91)	1546	44 (2.85)	1503	1 (0.07)
**≥60 years**						
<30 (deficiency)	170	19 (11.2)	164	13 (7.93)	157	6 (3.82)
30–50 (insufficiency)	637	72 (11.3)	624	59 (9.46)	578	13 (2.25)
≥50 (sufficiency)	1717	243 (14.2)	1686	212 (11.5)	1505	31 (2.06)

AMD, age-related macular degeneration.

**Table 3:** Odds ratios and 95% confidence intervals for AMD by serum 25(OH)D level according to the Institute of Medicine standard.

Serum 25(OH)D Level (nmol/L)	Any AMD			Early AMD			Late AMD		
	Crude	Model 1	Model 2	Crude	Model 1	Model 2	Crude	Model 1	Model 2
<30 (deficiency)	1.00 (ref)	1.00 (ref)	1.00 (ref)	1.00 (ref)	1.00 (ref)	1.00 (ref)	1.00 (ref)	1.00 (ref)	1.00 (ref)
30–50 (insufficiency)	1.22 (0.75–1.96)	1.08 (0.64–1.85)	1.07 (0.63–1.85)	1.44 (0.83–2.50)	1.34 (0.74–2.45)	1.33 (0.73–2.43)	0.61 (0.23–1.62)	0.33 (0.10–1.09)	0.27 (0.08–0.96)
≥50 (sufficiency)	1.61 (1.03–2.51)	1.21 (0.73–2.02)	1.17 (0.70–1.97)	1.96 (1.17–3.29)	1.54 (0.87–2.75)	1.50 (0.84–2.68)	0.65 (0.27–1.57)	0.34 (0.12–1.01)	0.28 (0.09–0.87)

Model 1: Adjusted for demographic variables (age, sex, race, education, poverty–income ratio) and lifestyle variables (body mass index, smoking, drinking). Model 2: Adjusted for demographic, lifestyle, medical comorbidity variables (general health condition, cardiovascular disease, diabetes, hypertension, hypercholesterolemia, history of cataract surgery, and glaucoma), and study cycle (2005–2006 vs. 2007–2008).

**Table 4:** Association of serum 25(OH)D status with AMD stratified by age.

Serum 25(OH)D (nmol/L)	Any AMD			Early AMD			Late AMD		
	Crude	Model 1	Model 2	Crude	Model 1	Model 2	Crude	Model 1	Model 2
**<60 years**									
<30 (deficiency)	1.00 (ref)	1.00 (ref)	1.00 (ref)	1.00 (ref)	1.00 (ref)	1.00 (ref)	1.00 (ref)	1.00 (ref)	1.00 (ref)
30–50 (insufficiency)	2.25 (0.67–7.56)	1.75 (0.50–6.07)	1.86 (0.53–6.50)	2.25 (0.67–7.56)	1.71 (0.49–5.95)	1.80 (0.51–6.32)	N.A.	N.A.	N.A.
≥50 (sufficiency)	2.14 (0.66–6.94)	1.96 (0.57–6.67)	1.82 (0.53–6.27)	2.09 (0.64–6.79)	1.82 (0.53–6.23)	1.67 (0.49–5.76)	N.A.	N.A.	N.A.
**≥60 years**									
<30 (deficiency)	1.00 (ref)	1.00 (ref)	1.00 (ref)	1.00 (ref)	1.00 (ref)	1.00 (ref)	1.00 (ref)	1.00 (ref)	1.00 (ref)
30–50 (insufficiency)	1.01 (0.59–1.73)	0.95 (0.52–1.74)	0.93 (0.50–1.70)	1.21 (0.65–2.27)	1.23 (0.61–2.45)	1.19 (0.60–2.39)	0.58 (0.22–1.55)	0.32 (0.09–1.08)	0.26 (0.07–0.93)
≥50 (sufficiency)	1.31 (0.80–2.15)	1.08 (0.61–1.91)	1.04 (0.59–1.86)	1.67 (0.93–3.00)	1.46 (0.75–2.81)	1.42 (0.73–2.77)	0.53 (0.22–1.29)	0.30 (0.10–0.91)	0.24 (0.08–0.75)

Model 1: Adjusted for demographic variables (age, sex, race, education, poverty–income ratio) and lifestyle variables (BMI, smoking, drinking). Model 2: Adjusted for demographic, lifestyle, medical comorbidity variables (general health condition, cardiovascular disease, diabetes, hypertension, hypercholesterolemia, history of cataract surgery, and glaucoma), and study cycle (2005–2006 vs. 2007–2008). N.A. not applicable.

## Data Availability

All data are publicly available at https://www.cdc.gov/nchs/nhanes/index.htm (accessed on 21 February 2023).

## Supplementary Materials

The following supporting information can be downloaded at: https://www.mdpi.com/article/10.3390/nu15051216/s1, Table S1: Comparison of demographic and general health factors of participants with or without 25(OH)D data. Table S2: Logistic regression models byserum 25(OH)D quintiles.
