# Supplementary material for: Serum 25-Hydroxyvitamin D Is Differentially Associated with Early and Late Age-Related Macular Degeneration in the United States Population
Source: Nutrients. 2023 Feb 28;15(5):1216. doi: 10.3390/nu15051216 (PMC10005371; doi:10.3390/nu15051216)
Supplement: Supplementary file 1 [file nutrients-15-01216-s001.zip › nutrients-2126513-supplementary.pdf]

Table S1. Comparison of demographic and general health factors of participants with or without vitamin D data

Variables	Participants with 25(OH)D data	Participants without 25(OH)D data	*P*-value
Sample size, n (%)	5041	563	
Participants with AMD, n (%)			0.380
No	4636 (92.0)	527 (93.6)	
Early	354 (7.0)	32 (5.7)	
Advanced	51 (1.0)	4 (0.7)	
Age, years, mean (SD)	59.6 (12.5)	58.4 (12.2)	0.036
Sex (female), n (%)	2504 (49.7)	289 (51.3)	0.455
Race/ethnicity, n (%)			
Mexican American	786 (15.6)	78 (13.9)	<0.001
Other Hispanic	345 (6.8)	56 (10.0)	
Non-Hispanic White	2791 (55.4)	226 (40.1)	
Non-Hispanic Black	966 (19.2)	173 (30.7)	
Other	153 (3.0)	30 (5.3)	
Education, n (%)			0.281
Less than 9th grade	720 (14.3)	81 (14.4)	
9-11th grade	744 (14.8)	98 (17.4)	
High school grad/GED or equivalent	1247 (24.7)	144 (25.6)	
Some college or AA degree	1286 (25.10	145 (25.8)	
College graduate or above	1043 (20.7)	95 (16.9)	
Poverty income ratio, n (%)			<0.001
< 130%	1168 (23.2)	159 (28.2)	
130 - 349%	1808 (35.9)	181 (32.2)	
≥ 350%	1745 (34.6)	147 (26.1)	
Unknown	320 (6.3)	76 (13.5)	
Obesity, n (%)			0.227
Underweight	67 (1.3)	12 (2.1)	
Normal	1214 (24.1)	148 (26.3)	
Overweight	1810 (35.9)	201 (35.7)	
Obese	1911 (37.9)	198 (35.2)	
Unknown	39 (0.8)	4 (0.7)	
Smoking status, n (%)			0.105
Non-smoker	2388 (47.4)	260 (46.2)	
Past smoker	1645 (32.6)	167 (29.7)	
Current smoker	1006 (19.9)	136 (24.2)	
Unknown	2 (0.1)	0	
Alcohol consumption, n (%)			<0.001
Never drinker	687 (13.6)	79 (14.0)	
Former drinker	536 (10.6)	54 (9.6)	
Current drinker	3715 (73.7)	395 (70.2)	
Unknown	103 (2.1)	35 (6.2)	

General health condition, n (%)			<0.001
Excellent, very good, or good	3702 (73.4)	355 (63.1)	
Fair, or poor	1251 (24.8)	176 (31.3)	
Unknown	88 (1.8)	32 (5.6)	
History of systemic diseases, n (%)			
Cardiovascular disease	770 (15.3)	94 (16.7)	0.376
Diabetes	1033 (20.5)	112 (19.9)	0.738
Hypertension	3364 (66.7)	370 (65.7)	0.629
Hypercholesterolemia	2084 (41.3)	196 (34.8)	<0.001
History of ocular condition, n (%)			
Cataract operation	633 (12.6)	64 (11.4)	0.572
Glaucoma	298 (5.9)	31 (5.5)	0.581

Table S2. Odds ratios and 95% confidence intervals for any and early AMD by quintiles of serum 25(OH)D level

Serum 25(OH)D (nmol/L)	Any AMD					Early AMD				
	No. at-risk	No. with event (%)	Crude	Model 1†	Model 2*	No. at-risk	No. with event (%)	Crude	Model 1†	Model 2*
**Overall**										
Q1 (<39.9)	1019	58 (5.69)	1.00 (ref)	1.00 (ref)	1.00 (ref)	1009	48 (4.76)	1.00 (ref)	1.00 (ref)	1.00 (ref)
Q2 (39.9-53.0)	998	74 (7.41)	1.32 (0.93-1.89)	1.16 (0.77-1.73)	1.15 (0.77-1.73)	988	64 (6.48)	1.39 (0.94-2.04)	1.25 (0.82-1.92)	1.24 (0.81-1.91)
Q3 (53.1-64.1)	1026	77 (7.50)	1.34 (0.95-1.91)	1.01 (0.67-1.52)	0.99 (0.65-1.50)	1013	64 (6.32)	1.35 (0.92-1.98)	1.02 (0.65-1.59)	1.00 (0.64-1.57)
Q4 (64.2-78.0)	993	97 (9.77)	**1.79 (1.28-2.51)**	1.45 (0.99-2.15)	1.42 (0.95-2.12)	986	90 (9.13)	**2.01 (1.40-2.89)**	**1.68 (1.11-2.55)**	**1.65 (1.08-2.52)**
Q5 (≥78.1)	1005	99 (9.85)	**1.81 (1.29-2.53)**	1.35 (0.91-2.02)	1.28 (0.85-1.94)	994	88 (8.85)	**1.94 (1.35-2.80)**	**1.54 (1.00-2.35)**	1.46 (0.94-2.27)
**< 60 years**										
Q1 (<39.9)	579	9 (1.55)	1.00 (ref)	1.00 (ref)	1.00 (ref)	579	9 (1.55)	1.00 (ref)	1.00 (ref)	1.00 (ref)
Q2 (39.9-53.0)	513	19 (3.70)	**2.43 (1.09-5.43)**	**2.72 (1.10-6.78)**	**2.69 (1.08-6.73)**	513	19 (3.70)	**2.43 (1.09-5.43)**	**2.66 (1.07-6.63)**	**2.60 (1.04-6.49)**
Q3 (53.1-64.1)	513	13 (2.53)	1.65 (0.70-3.88)	2.16 (0.82-5.71)	2.06 (0.77-5.47)	513	12 (2.34)	1.52 (0.64-3.64)	1.91 (0.71-5.14)	1.80 (0.67-4.86)
Q4 (64.2-78.0)	460	17 (3.70)	**2.43 (1.07-5.50)**	**3.06 (1.18-7.91)**	**2.78 (1.06-7.27)**	460	17 (3.70)	**2.43 (1.07-5.50)**	**2.94 (1.14-7.61)**	**2.64 (1.01-6.93)**
Q5 (≥78.1)	452	13 (2.88)	1.88 (0.79-4.43)	2.12 (0.77-5.86)	1.56 (0.54-4.54)	452	13 (2.88)	1.88 (0.79-4.43)	2.04 (0.74-5.64)	1.48 (0.51-4.30)
**≥ 60 years**										
Q1 (<39.9)	440	49 (11.1)	1.00 (ref)	1.00 (ref)	1.00 (ref)	430	39 (9.07)	1.00 (ref)	1.00 (ref)	1.00 (ref)
Q2 (39.9-53.0)	485	55 (11.3)	1.02 (0.68-1.54)	0.91 (0.58-1.44)	0.90 (0.57-1.43)	475	45 (9.47)	1.05 (0.67-1.65)	0.97 (0.59-1.59)	0.96 (0.58-1.58)
Q3 (53.1-64.1)	513	64 (12.5)	1.14 (0.77-1.69)	0.82 (0.52-1.30)	0.81 (0.51-1.29)	501	52 (10.4)	1.16 (0.75-1.80)	0.84 (0.51-1.39)	0.83 (0.50-1.38)
Q4 (64.2-78.0)	533	80 (15.0)	1.41 (0.96-2.06)	1.21 (0.78-1.88)	1.19 (0.76-1.86)	526	73 (13.9)	**1.62 (1.07-2.44)**	1.42 (0.89-2.27)	1.42 (0.88-2.28)
Q5 (≥78.1)	553	86 (13.2)	**1.46 (1.01-2.14)**	1.19 (0.76-1.86)	1.18 (0.75-1.86)	542	75 (13.8)	**1.61 (1.07-2.43)**	1.37 (0.86-2.21)	1.38 (0.85-2.24)

†Model 1: Adjusted for demographic variables (age, sex, race, education, poverty income ratio) and lifestyle variables (BMI, smoking, drinking). * Model 2: Adjusted for demographic, lifestyle, medical comorbidity variables (general health condition, cardiovascular disease, diabetes, hypertension, hypercholesterolemia, history of cataract surgery, and glaucoma) and study cycle (2005-2006 vs. 2007-2008).
